# Analytical sensitivity factors from distributions of time of flight of photons for near-infrared spectroscopy studies in multilayered turbid media

**DOI:** 10.1117/1.JBO.30.1.015002

**Published:** 2025-01-22

**Authors:** Héctor A. García, Demián A. Vera, Nicolás A. Carbone, María V. Waks-Serra, Juan A. Pomarico

**Affiliations:** a University of Wisconsin–Madison, School of Medicine and Public Health, Department of Medical Physics, Madison, Wisconsin, United States; b CIFICEN (UNCPBA - CICPBA - CONICET), Tandil, Argentina

**Keywords:** near-infrared spectroscopy, multilayered media, sensitivity factors, distributions of times of flight, diffusion theory, Monte Carlo

## Abstract

**Significance:**

In the last years, time-resolved near-infrared spectroscopy (TD-NIRS) has gained increasing interest as a tool for studying tissue spectroscopy with commercial devices. Although it provides much more information than its continuous wave counterpart, accurate models interpreting the measured raw data in real time are still lacking.

**Aim:**

We introduce an analytical model that can be integrated and used in TD-NIRS data processing software and toolkits in real time. This is based on the so-called sensitivity factors (SFs) of the distributions of time of flight (DTOFs) of photons measured in optically turbid and semi-infinite multilayered media, such as the human head.

**Approach:**

We derived analytical expressions for the SFs that link changes in the absorption coefficient of each layer to changes in the statistical moments of DTOFs acquired in a reflectance configuration. This was later validated with results from Monte Carlo (MC) simulations, which stand as the gold standard in terms of photon migration in biological tissue. Next, we designed a couple of simulated experiments depicting how the analytical SFs can be used to retrieve absorption changes in the particular case of a five-layered medium.

**Results:**

Comparison between theory and simulations in 2-, 5-, and 10-layered media showed very good agreement (in most cases with weighted mean absolute percentage errors below 10%). Moreover, our derivations could be run in a few milliseconds (except for the extreme case of the variance SF in the 10-layered medium), which means a speedup of up to 10,000× with respect to MC simulations, with a much better spatial resolution and without their typically associated stochastic noise.

**Conclusions:**

In summary, our method achieves performances similar to those given by MC simulations, but orders of magnitude faster, which makes it very suitable for its implementation in real-time applications.

## 1 Introduction

Functional near-infrared spectroscopy (fNIRS) is a technique that aims at optically characterizing brain hemodynamics in a non-invasive way by means of non-expensive and portable devices and components.^1^^,^^2^ Because of this, fNIRS has found numerous applications in neurology and neuroscience, such as the study of neurological disorders,^3^ sports and movement sciences,^4^^,^^5^ and speech development in newborns and children.^6^ Its principle of working relies on the use of NIR light sources (between 630 and 900 nm) placed on the external surface of the tissue to be studied, together with a set of detector fibers appropriately placed at specific distances from the sources, which are used to collect the diffusely reflected light. This detected signal carries information about the optical characteristics of the medium—i.e., the absorption coefficient, $\mu_{a}$, and the reduced scattering coefficient, $\mu_{s}^{\prime }$—and, consequently, about biochemical constituents such as hemoglobin, glucose, and oxygen.

The different types of existing fNIRS devices can be classified into two main categories: continuous wave (CW) and time-resolved (TR) systems.^1^^,^^7^ The CW approach is the most widespread because it allows for the design of more inexpensive, compact, and easy-to-use devices. On the other hand, TR devices are usually more expensive and cumbersome, but they can also provide much more information with a reduced number of sources and detectors,^7^^–^^9^ being one of the reasons why TR commercial devices have been reaching the market in the last few years.^8^^,^^10^^–^^12^ Time-resolved NIRS systems send ultrashort light pulses which travel through tissue and then are collected by detectors with very high time resolution in the form of distributions of times of flight (DTOFs) of photons. The difference in shape between the DTOFs and the incoming pulses is related to the optical properties of the tissue; in addition, in this case, depth discrimination can be performed by splitting the DTOFs into different time windows where the signal can be classified into early- or late-arrival photons^13^^,^^14^ and also by analyzing the statistical DTOF moments.^15^ This second approach is particularly advantageous because the influence of the instrument response function (IRF) characterizing the measuring system can be ignored.

Previous works have shown that the higher the order of the DTOF moment, the higher the sensitivity to absorption changes in deeper regions of the studied medium.^16^^–^^18^ This fact is especially important when considering living tissue where hemodynamic changes do not occur homogeneously throughout the whole volume, but rather localized in specific subregions. This is the case of the human head, which can be considered as a stack of layers (such as the scalp, skull, cerebrospinal fluid, and gray matter) surrounding the white matter. Here, each layer can present its own change in blood flow, and consequently, the amount of light absorbed at different depths is not the same; in particular, brain activation targets hemodynamic changes in the cortex, so determining to what extent the measured light signal is contaminated by the influence of the remaining layers is of capital importance.

Reconstruction of light absorption changes in the human head using TR techniques can be performed by means of the so-called sensitivity factors (SFs) of DTOFs.^16^^,^^17^ This method, which has proved to be very accurate, has been implemented so far mostly with the help of Monte Carlo (MC) simulations. MC simulations are the gold standard for modeling photon migration in complex turbid media of arbitrary geometries;^19^^,^^20^ however, the very high computation times and sometimes prohibitive hardware requirements usually associated with MC simulations definitely prevent them from being used in real-time applications. Hence, faster and more economical implementations are needed.

In this work, we introduce the derivation of analytical expressions for the SFs to retrieve absorption changes in turbid media consisting of an arbitrary number of layers. These expressions arise after approximating the radiative transfer equation with the diffusion equation, which is restricted to turbid media and optode configurations compatible with a diffuse regime, where a scattering of light is strong when compared with absorption.^21^ Although there exist previous attempts of doing this, they rely on heuristic methods;^22^^–^^24^ on the other hand, we propose a formal derivation by fully departing from analytical models of light propagation in multilayered turbid media without any additional approximations. To validate our model, we compare the analytical results with the outcomes from Monte Carlo simulations, showing that differences between both methods never exceed 10% (except for the upper layer in a few situations), thus demonstrating that the analytical approach has a performance similar to MC simulations, but up to four orders of magnitude faster. Then, we show a few application examples depicting how the SFs can be used to retrieve changes in the absorption coefficients in real-case scenarios such as a five-layered medium mimicking the tissue layers into which the human head is usually segmented. Last, we provide full access to the codes for the analytical expressions introduced here.^25^

This article is structured as follows. In Sec. 2, we introduce our analytical model and the most important steps for its derivation. Sec. 3 presents the details on the MC code used in this work. Sec. 4 shows the results of comparing the SFs obtained by both methods. Finally, in Sec. 5, we state the main conclusions and discuss possible future applications.

## 2 Methods

**Fig. 1:**
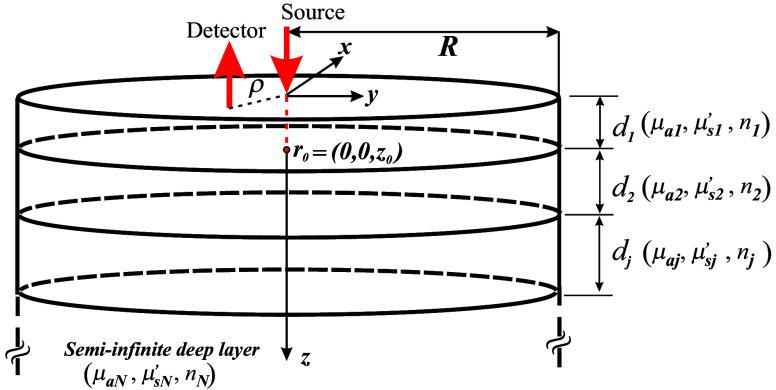
Scheme of the multilayered turbid cylinder used for the derivation of the analytical expressions introduced in this section. The pencil beam–like source is physically placed at r=(0,0,0), and light propagation becomes isotropic at a position $r_{0}=(0,0,z_{0})$, where $z_{0}=1/\mu_{s1}^{\prime }$.

Consider an optically turbid cylinder of radius R consisting of N different layers (Fig. 1), being the j’th layer characterized by its absorption coefficient $\mu_{\mathrm{aj}}$, reduced scattering coefficient $\mu_{\mathrm{sj}}^{\prime }$, refractive index $n_{j}$, and thickness $d_{j}$ (except for the last layer, for which $d_{N}\to \infty$). According to the diffusion approximation (DA),^26^ the time-resolved diffuse reflectance R(ρ,t) measured at time t and at a distance ρ from a pencil beam–like source placed at the center of the cylinder’s upper surface and impinging downward can be analytically expressed as follows:

$$R(\rho ,t)=\frac{1}{4\pi^{2}AR_{\mathrm{EB}}}\overset{\infty }{\underset{n=1}{\sum }}\frac{J_{0}(s_{n}\rho )}{J_{1}^{2}(s_{n}R_{\mathrm{EB}})}\int_{-\infty }^{\infty }G_{1}(z=0,\omega ,s_{n})e^{i\omega t}d\omega ,$$ (1)

where A is a factor accounting for the differences in the external and the medium’s refractive indices;^27^ $R_{\mathrm{EB}}$ is the extrapolated radius where the reflectance vanishes (according to the extrapolated boundary condition^28^); $J_{0}$ and $J_{1}$ are the Bessel functions of the first kind and of orders zeroth and first, respectively; $G_{1}$ is Green’s function of the first layer; ω is the modulation frequency; and $s_{n}$ is such that $J_{0}(s_{n}R_{\mathrm{EB}})=0$.

Green’s function in Eq. (1) takes the form

$$G_{1}(z=0,\omega ,s_{n})=\frac{e^{-\alpha_{1}z_{0}}-e^{-\alpha_{1}(z_{0}+2z_{b})}}{2D_{1}\alpha_{1}}+\frac{\sin\;h[\alpha_{1}(z_{0}+z_{b})]\sin\;h[\alpha_{1}(z_{b})]}{D_{1}\alpha_{1}e^{d_{1}+z_{b}}}\times \;\times \frac{D_{1}\alpha_{1}n_{1}^{2}\beta_{3}-D_{2}\alpha_{2}n_{2}^{2}\gamma_{3}}{D_{1}\alpha_{1}n_{1}^{2}\beta_{3}\;\cos\;h[\alpha_{1}(d_{1}+z_{b})]+D_{2}\alpha_{2}n_{2}^{2}\gamma_{3}\;\sin\;h[\alpha_{1}(d_{1}+z_{b})]}.$$ (2)

Here, $\alpha_{j}=\sqrt{\mu_{aj}/D_{j}+s_{n}^{2}+i\omega /(D_{j}c_{j})}$, $D_{j}=(3\mu_{sj}^{\prime }{)}^{-1}$ is the diffusion coefficient in the j’th layer, $c_{j}$ is the speed of light in that same layer, i is the imaginary unit, and $z_{b}$ is the extrapolated surface in the z-direction. Regarding the quantities $\beta_{3}$ and $\gamma_{3}$, they are obtained by means of recursion relations with initial values $\beta_{N}$ and $\gamma_{N}$, given by

$$\beta_{N}=D_{N-1}\alpha_{N-1}n_{N-1}^{2}\;\cos\;h(\alpha_{N-1}d_{N-1})+D_{N}\alpha_{N}n_{N}^{2}\;\sin\;h(\alpha_{N-1}d_{N-1}),\;\gamma_{N}=D_{N-1}\alpha_{N-1}n_{N-1}^{2}\;\sin\;h(\alpha_{N-1}d_{N-1})+D_{N}\alpha_{N}n_{N}^{2}\;\cos\;h(\alpha_{N-1}d_{N-1}),$$ (3)

and then going “all the way down” to k=4 as follows:

$$\beta_{k-1}=D_{k-2}\alpha_{k-2}n_{k-2}^{2}\;\cos\;h(\alpha_{k-2}d_{k-2})\beta_{k}+D_{k-1}\alpha_{k-1}n_{k-1}^{2}\;\sin\;h(\alpha_{k-2}d_{k-2})\gamma_{k},\;\gamma_{k-1}=D_{k-2}\alpha_{k-2}n_{k-2}^{2}\;\sin\;h(\alpha_{k-2}d_{k-2})\beta_{k}+D_{k-1}\alpha_{k-1}n_{k-1}^{2}\;\cos\;h(\alpha_{k-2}d_{k-2})\gamma_{k}.$$ (4)

For this study, we set R=250  mm and used up to 5000 Bessel zeros; in the end, convergence is ruled by the combination of these parameters with the optical properties and thickness of each layer (detailed in Sec. 3).

**Fig. 2:**
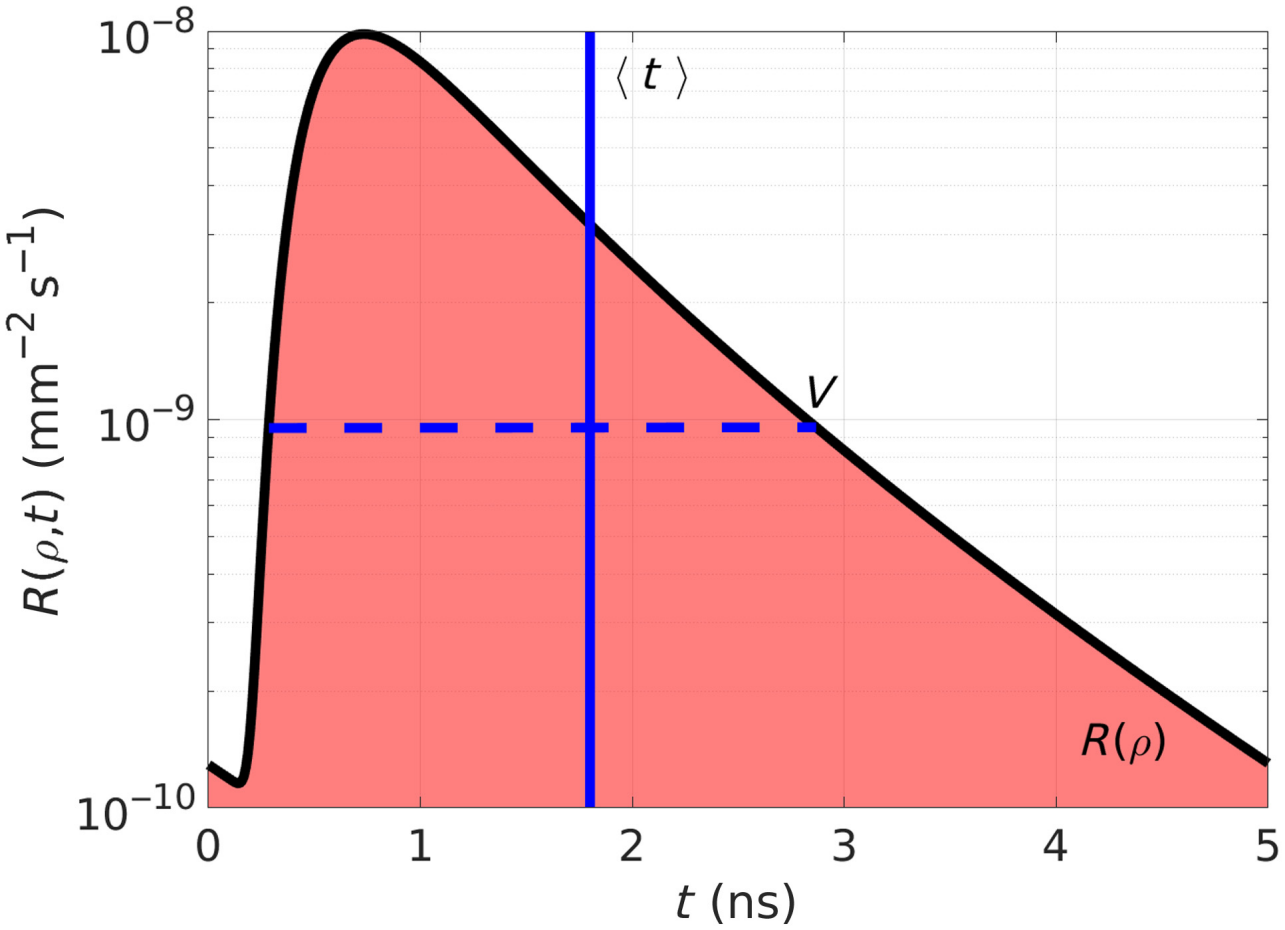
Example of DTOF (black line) generated with expression Eq. (1) for a two-layered medium with optical properties $(\mu_{a1},\mu_{a2})=(0.01,0.002)\;{\mathrm{mm}}^{-1}$, $(\mu_{s1}^{\prime },\mu_{s2}^{\prime })=(0.9,1.1)\;{\mathrm{mm}}^{-1}$, and $n_{1}=n_{2}=1.33$; the thickness of the first layer is $d_{1}=10\;\mathrm{mm}$, and the source–detector distance is ρ=30  mm. In addition, the integrated DTOF as a function of ρ, R(ρ), has been schemed as the light red area under the DTOF, together with the mean time of flight ⟨t⟩ (blue vertical line) and the variance V (blue horizontal dashed line).

Figure 2 shows an example of a theoretical DTOF computed with the help of Eq. (1) as a function of time. In addition, in this figure, we schematized the DTOF’s first three statistical moments, namely, the total number of photons counts, which is nothing else than the CW diffuse reflectance R(ρ) (given by the area under the curve); the mean time of flight ⟨t⟩ (blue vertical line); and the second-order centralized moment, i.e., the variance V (blue horizontal dashed line). Now, in applications such as fNIRS, in general, it is assumed that changes in hemoglobin concentrations in the blood have an impact only on the absorption coefficient of the tissue, whereas the reduced scattering coefficient remains practically constant.^29^^,^^30^ Under this assumption, it is possible to express the changes in the DTOF’s moments as functions of the absorption changes in each layer

$$\Delta R(\rho )\to L(\rho )\cdot \Delta \mu_{a}^{T},\;\Delta \langle t\rangle (\rho )=\mathrm{MTSF}(\rho )\cdot \Delta \mu_{a}^{T},\;\Delta V(\rho )=\mathrm{VSF}(\rho )\cdot \Delta \mu_{a}^{T},$$ (5)

where $\Delta \mu_{a}=(\Delta \mu_{a1},\Delta \mu_{a2},\ldots ,\Delta \mu_{aN})$ is the vector of absorption changes in each layer (with the superscript T denoting transposition), $L(\rho )=(L_{1},L_{2},\ldots ,L_{N})(\rho )$ is the vector of mean partial pathlengths (MPPLs) of photons, $\mathrm{MTSF}(\rho )=({\mathrm{MTSF}}_{1},{\mathrm{MTSF}}_{2},\ldots ,{\mathrm{MTSF}}_{N})(\rho )$ is the vector of mean time sensitivity factors (MTSFs), and $\mathrm{VSF}(\rho )=({\mathrm{VSF}}_{1},{\mathrm{VSF}}_{2},\ldots ,{\mathrm{VSF}}_{N})(\rho )$ is the vector of variance sensitivity factors (VSFs). In the following, general expressions for the MPPLs, MTSFs, and VSFs involving the diffuse reflectance will be derived (the reason for the right arrow instead of the equal sign in the first entry of Eq. (5) will be clear in the Sec. 2.1); in addition, in the accompanying Supplementary Material, we also provide more specific results for the particular case of semiinfinite multilayered turbid media.

### 2.1 Mean Partial Pathlengths

Let us assume a DTOF measurement on an optically dynamic multilayered turbid medium (i.e., its optical properties vary with time, particularly $\mu_{aj}$). If we are able to refer the current measurement R(ρ) to a baseline level $R_{b}(\rho )$, then we can express the first relation in Eq. (5) in terms of the attenuation change ΔA(ρ)^31^

$$\Delta A(\rho )=-\log[\frac{R(\rho )}{R_{b}(\rho )}]\equiv -\log[\frac{R(\rho )}{R_{0}}]+\log[\frac{R_{b}(\rho )}{R_{0}}]=A(\rho )-A_{b}(\rho ),$$ (6)

where $R_{0}$ is the signal from the source, and ΔA(ρ) is change in the attenuation being measured with respect to the baseline value $A_{b}(\rho )$. The last two members of Eq. (6) allow us to study the change in attenuation caused by a change in the absorption of the j’th layer as follows:

$$\Delta A(\rho )=\frac{\partial A_{b}(\rho )}{\partial \mu_{aj}}\Delta \mu_{aj}.$$ (7)

Now, combining Eqs. (6) and (7), we can reach the next relation

$$\Delta A(\rho )=-\frac{1}{R_{b}(\rho )}\frac{\partial R_{b}(\rho )}{\partial \mu_{aj}}\Delta \mu_{aj}.$$ (8)

The quantity

$$-\frac{1}{R_{b}(\rho )}\frac{\partial R_{b}(\rho )}{\partial \mu_{aj}}=L_{j}(\rho ),$$ (9)

is none other than the previously defined MPPL of photons in the j’th layer and can be considered as the sensitivity factor that links attenuation (and, consequently, the 0’th order moment of R(ρ)) and an absorption change in that specific layer.^32^^–^^35^ Expression Eq. (8) has been derived under the assumption that the absorption change takes place only in one layer; a more general expression considering changes in every layer of an N-layered turbid medium can be straightforwardly derived, and has the following form:^36^^,^^37^

$$\Delta A(\rho )=L(\rho )\cdot \Delta \mu_{a}^{T}=\overset{N}{\underset{j=1}{\sum }}L_{j}(\rho )\Delta \mu_{\mathrm{aj}}.$$ (10)

The spirit of this last relation is the same as the first one in Eq. (5), but now with the equal sign instead of the right arrow.

Equation (9) tells that the derivative of R(ρ) with respect to $\mu_{\mathrm{aj}}$ is needed to compute $L_{j}(\rho )$. The corresponding calculations will be omitted here because they were already done for two-, three- and four-layered media on one side^34^ and for an arbitrary number of layers on the other.^35^^,^^38^ Nevertheless, further details can be found in the Supplementary Material accompanying this article.

As a closure for this section, we additionally provide a way of analytically obtaining the so-called time-dependent MPPLs,^16^^,^^17^ which can basically be computed by replacing R(ρ) in Eq. (9) by its time-domain counterpart

$$L_{j}(\rho ,t)=-\frac{1}{R_{b}(\rho ,t)}\frac{\partial R_{b}(\rho ,t)}{\partial \mu_{aj}}.$$ (11)

### 2.2 Mean Time of Flight Sensitivity Factor

Similar to what was done before for the change in attenuation, here, we can express the change in the mean time of flight, Δ⟨t⟩, as a Taylor expansion

$$\Delta \langle t\rangle (\rho )=\nabla_{\mu_{a}}\langle t\rangle (\rho )\cdot \Delta \mu_{a}^{T}=\overset{N}{\underset{j=1}{\sum }}\frac{\partial \langle t\rangle (\rho )}{\partial \mu_{aj}}\Delta \mu_{aj},$$ (12)

where the subscript $\mu_{a}$ in the gradient operator indicates that the derivatives are taken with respect to each $\mu_{\mathrm{aj}}$. Comparing this expression with the second entry in Eq. (5), it is possible to define the mean time sensitivity factor in layer j, ${\mathrm{MTSF}}_{j}(\rho )$, as

$${\mathrm{MTSF}}_{j}(\rho )=\frac{\partial \langle t\rangle (\rho )}{\partial \mu_{aj}}.$$ (13)

The next step is to rewrite ⟨t⟩(ρ) in terms of $R_{b}(\rho )$. To this end, let us first split ⟨t⟩(ρ) into the sum of the mean partial times that photons spend in each layer

$$\langle t\rangle (\rho )=\overset{N}{\underset{k=1}{\sum }}\langle t_{k}\rangle (\rho )=\overset{N}{\underset{k=1}{\sum }}\frac{L_{k}(\rho )}{c_{k}}.$$ (14)

Then, Eq. (13) takes the form

$${\mathrm{MTSF}}_{j}(\rho )=\overset{N}{\underset{k=1}{\sum }}\frac{1}{c_{k}}\frac{\partial L_{k}(\rho )}{\partial \mu_{aj}}.$$ (15)

Hence, we need the derivatives of $L_{k}(\rho )$ with respect to $\mu_{aj}$

$$\frac{\partial L_{k}(\rho )}{\partial \mu_{aj}}=\frac{\partial }{\partial \mu_{aj}}[-\frac{1}{R_{b}(\rho )}\frac{\partial R_{b}(\rho )}{\partial \mu_{ak}}]=\frac{1}{R_{b}^{2}(\rho )}\frac{\partial R_{b}(\rho )}{\partial \mu_{aj}}\frac{\partial R_{b}(\rho )}{\partial \mu_{ak}}-\frac{1}{R_{b}(\rho )}\frac{\partial^{2}R_{b}(\rho )}{\partial \mu_{aj}\partial \mu_{ak}},$$ (16)

or by means of Eq. (9),

$$\frac{\partial L_{k}(\rho )}{\partial \mu_{aj}}=L_{j}(\rho )L_{k}(\rho )-\frac{1}{R_{b}(\rho )}\frac{\partial^{2}R_{b}(\rho )}{\partial \mu_{aj}\partial \mu_{ak}}.$$ (17)

Here, we will omit further details for the sake of brevity but encourage the reader to find them in the Supplementary Material accompanying this work.

### 2.3 Variance Sensitivity Factor

Once again, we can Taylor expand the change in the variance as follows:

$$\Delta V(\rho )=\nabla_{\mu_{a}}V\cdot \Delta \mu_{a}^{T}=\overset{N}{\underset{j=1}{\sum }}\frac{\partial V(\rho )}{\partial \mu_{aj}}\Delta \mu_{aj}.$$ (18)

Therefore, the variance sensitivity factor in layer j, ${\mathrm{VSF}}_{j}(\rho )$ can be written as

$${\mathrm{VSF}}_{j}(\rho )=\frac{\partial V(\rho )}{\partial \mu_{aj}}.$$ (19)

Now, expressing V(ρ) in terms of $R_{b}(\rho )$ requires rewriting the variance in the well-known fashion,

$$V(\rho )=\langle t^{2}\rangle (\rho )-\langle t\rangle^{2}(\rho ).$$ (20)

Deriving Eq. (20) with respect to $\mu_{aj}$ leads us to

$$\frac{\partial V(\rho )}{\partial \mu_{aj}}=\frac{\partial \langle t^{2}\rangle (\rho )}{\partial \mu_{aj}}-2\langle t\rangle (\rho )\frac{\partial \langle t\rangle (\rho )}{\partial \mu_{aj}}.$$ (21)

The last term of this expression can be easily rewritten with the help of Eqs. (13) and (14) as

$$-2\langle t\rangle \frac{\partial \langle t\rangle }{\partial \mu_{aj}}=-2\overset{N}{\underset{k=1}{\sum }}\frac{L_{k}(\rho )}{c_{k}}{\mathrm{MTSF}}_{j}(\rho ).$$ (22)

On the other hand, the first term in expression Eq. (21) requires some more attention. First, it is convenient to begin with the fact that $t=\sum_{j=1}^{N}t_{j}$; then

$$\langle t^{2}\rangle =\langle \overset{N}{\underset{k=1}{\sum }}\overset{N}{\underset{l=1}{\sum }}t_{k}t_{l}\rangle =\overset{N}{\underset{k=1}{\sum }}\overset{N}{\underset{l=1}{\sum }}\langle t_{k}t_{l}\rangle .$$ (23)

Next, we can make use of the following facts: (i) the first moment of $t_{j}$, $\left\langle t_{j}\right\rangle$, can be expressed as a function of the derivative of $R_{b}$ with respect to $\mu_{aj}$ [this is given, basically, by expression Eq. (9)], and (ii) the second moment of t (for a homogeneous medium), $\left\langle t^{2}\right\rangle$, can be computed as^39^

$$\langle t^{2}\rangle =\frac{1}{c^{2}R_{b}(\rho )}\frac{\partial^{2}R_{b}(\rho )}{\partial \mu_{aj}^{2}}.$$ (24)

Combining these two relations, the expectation value $\left\langle t_{k}t_{l}\right\rangle$ in Eq. (23) can be rewritten in the following way:

$$\langle t_{k}t_{l}\rangle =\frac{1}{c_{k}c_{l}R_{b}(\rho )}\frac{\partial^{2}R_{b}(\rho )}{\partial \mu_{ak}\partial \mu_{al}}.$$ (25)

Now, we are finally in the condition of handling the first term of Eq. (21),

$$\frac{\partial \langle t^{2}\rangle }{\partial \mu_{aj}}=\overset{N}{\underset{k=1}{\sum }}\overset{N}{\underset{l=1}{\sum }}\frac{\partial \langle t_{k}t_{l}\rangle }{\partial \mu_{aj}}=\overset{N}{\underset{k=1}{\sum }}\overset{N}{\underset{l=1}{\sum }}\frac{1}{c_{k}c_{l}}\frac{1}{R_{b}(\rho )}\times [\frac{\partial^{3}R_{b}(\rho )}{\partial \mu_{aj}\partial \mu_{ak}\partial \mu_{al}}-\frac{1}{R_{b}(\rho )}\frac{\partial R_{b}(\rho )}{\partial \mu_{aj}}\frac{\partial^{2}R_{b}(\rho )}{\partial \mu_{ak}\partial \mu_{al}}].$$ (26)

It is important to notice that reaching Eq. (26) required assuming the validity of relation Eq. (25), which has been derived in a rather heuristic fashion. Nevertheless, in the Sec. 3.1, we provide further evidence of this; in addition, and as it will be shown in the results, the matching between the analytical VSFs and the ones computed with MC simulations suggests that Eq. (25) fully holds.

## 3 Monte Carlo Simulations

Monte Carlo simulations stand as the gold standard method for light propagation in turbid and semitransparent media, especially because it does not suffer from the limitations imposed by the diffusion approximation.^19^ In this work, MC simulations were employed (i) to compute $L_{j}$, ${\mathrm{MTSF}}_{j}$, and ${\mathrm{VSF}}_{j}$ for different combinations of optical and geometrical parameters in media with different amounts of layers, and then these outputs were compared with the corresponding analytical expressions Eqs. (9), (13), and (19) and (ii) to show a set of examples of how the SFs can be used to retrieve $\mu_{a}$ variations in real-world cases.

### 3.1 Validation

To validate our method, we used MCXlab, a MATLAB-based toolbox for MC simulations in turbid media.^40^^,^^41^ Every simulation consisted of launching $10^{10}$ photons through a pencil beam–like source (the same as in the analytical model) in a voxelized turbid medium of volume $500\times 500\times 500\;{\mathrm{mm}}^{3}$ (voxel size: $1\;{\mathrm{mm}}^{3}$), split into layers with their own set of optical properties and thicknesses (except for the last one, which was semi-infinite). Details on the type of simulated media and their optical and geometrical characteristics can be found in Table 1. All the simulations were run in a workstation with an AMD^®^ Ryzen 9 7950× 16-core processor with a NVIDIA GeForce RTX 4090 graphics processing unit (GPU).

**Table 1:** Optical and geometrical parameters considered for both the MC simulations and the analytical computations of the MPPLs, MTSFs, and VSFs. These values were taken from Refs. 42 and 43. Unless otherwise stated, the refractive index and the anisotropy factor of each layer were set to $n_{j}=1.33$ and $g_{j}=0.9$, respectively.

Medium	$\mu_{a}$ ($10^{-2}\;{\mathrm{mm}}^{-1}$)	$\mu_{s}^{\prime }$ (${\mathrm{mm}}^{-1}$)	d (mm)
2-layered	(1.8 and 1.7)	(1.9 and 2.3)	5
5-layered	(1.8, 1.6, 0.2, 3.6, and 1.4)	(1.9, 1.6, 1, 2.2, and 4.1)	(5, 5, 2, and 4)
10-layered	(0.2, 2, 1, 1.60, 0.4, 0.8, 1.4, 1.8, 1.2, and 0.6)	(2.0, 1.12, 0.81, 0.50, 0.88, 0.96, 0.73, 1.04, 0.57, and 0.65)	(2, 2, 2, 2, 2, 2, 2, 2, and 2)

Finally, it is instructive to provide a comparison among the expressions needed to compute the sensitivity factors by means of MC simulations and the analytical ones derived in this publication. According to Liebert et al.,^17^ the MC MPPLs can be calculated as follows:

$$L_{j}(\rho )=c_{j}\langle t_{j}\rangle (\rho )=\frac{\underset{i}{\sum }l_{ij}W_{i}}{\underset{i}{\sum }W_{i}},$$ (27)

being $l_{ij}$ the partial pathlength of the detected photon i, an $W_{i}$ its corresponding weight at the moment of the detection process. This expression is equivalent to Eq. (9)

In the case of the MTSF, the MC version is

$${\mathrm{MTSF}}_{j}(\rho )=-\overset{N}{\underset{m=1}{\sum }}\frac{\left\langle l_{j}l_{m}\rangle (\rho \right)}{c_{m}}+L_{j}(\rho )\langle t\rangle (\rho ),$$ (28)

with the cross term given by

$$\langle l_{j}l_{m}\rangle =\frac{\underset{i}{\sum }l_{ij}l_{im}W_{i}}{\underset{i}{\sum }W_{i}}.$$ (29)

The analytical counterpart of Eq. (28) is Eq. (13).

Lastly, the MC version of the VSF takes the form

$${\mathrm{VSF}}_{j}(\rho )=-\overset{N}{\underset{m=1}{\sum }}\overset{N}{\underset{n=1}{\sum }}\frac{\left\langle l_{j}l_{m}l_{n}\rangle (\rho \right)}{c_{m}c_{n}}++2\langle t\rangle (\rho )\overset{N}{\underset{m=1}{\sum }}\frac{\left\langle l_{j}l_{m}\rangle (\rho \right)}{c_{m}}+L_{j}(\rho )[\langle t^{2}\rangle (\rho )-2\langle t\rangle^{2}(\rho )],$$ (30)

where the triple cross product is

$$\langle l_{j}l_{m}l_{n}\rangle (\rho )=\frac{\underset{i}{\sum }l_{ij}l_{im}l_{\text{in}}W_{i}}{\underset{i}{\sum }W_{i}}.$$ (31)

The analytical equation matching this last expression is Eq. (19).

A closer inspection to these equivalences makes us notice that, whenever an expected value ⟨·⟩ in the MC version arises, a derivative appears in the analytical version. In addition, the order of the analytical derivative increases with the amount of arguments in the MC expectation values; this can be directly seen by comparing Eq. (9) with Eq. (27), or more subtly by comparing the last term in Eq. (17) with the first term in Eq. (28). In that sense, the triple cross-product given by relation Eq. (31) can be associated to the first term in the squared brackets of expression Eq. (26). This is the reason to assume that Eq. (25) is valid.

### 3.2 Application Examples

To show the capabilities of the SFs, we emulated absorption changes using MC simulations on a medium consisting of five layers (the last one being semi-infinite) with the same geometrical and (initial) optical properties as the five-layered medium considered for the validation of the SFs (see Table 1). To mimic a realistic process, these changes were introduced in the first and fourth layers, which play the role of the scalp and gray matter, respectively.^36^^,^^37^ The raw signals $\Delta M(\rho_{1})$ and $\Delta M(\rho_{2})$ (where ΔM can be either ΔA, Δ⟨t⟩, or Δ⟨V⟩) were obtained with the diffuse reflectance data calculated from the MC simulations. Then, the desired absorption changes were retrieved by inverting the following matrix equation:

$$[\begin{array}{c} \Delta M(\rho_{1})\\ \Delta M(\rho_{2}) \end{array}]=[\begin{array}{cc} {\mathrm{SF}}_{U}(\rho_{1}) & {\mathrm{SF}}_{L}(\rho_{1})\\ {\mathrm{SF}}_{U}(\rho_{2}) & {\mathrm{SF}}_{L}(\rho_{2}) \end{array}][\begin{array}{c} \Delta \mu_{aU}\\ \Delta \mu_{aL} \end{array}].$$ (32)

Here, SF denotes either MPPL, MTSF, or VSF (as given by Eqs. (9), (13), and (19), respectively), and the subscripts U and L correspond to the upper (first) layer and the lower (fourth) layer (i.e., only those layers where the absorption changes take place), respectively.

As shown before,^36^^,^^37^ combining two different source–detector separations allows us to retrieve changes in absorption in two different layers. To this end, we considered two different pairs of source–detector distances, $(\rho_{1},\rho_{2})=(5,15)\;\mathrm{mm}$ and $(\rho_{1},\rho_{2})=(10,30)\;\mathrm{mm}$, and then we analyzed the impact of each choice in the reconstructed data. So far, this has been done analytically only using the MPPLs, but here, we are in conditions to retrieve the desired quantities also with the help of the remaining SFs.

## 4 Results

### 4.1 Validation Results

**Fig. 3:**
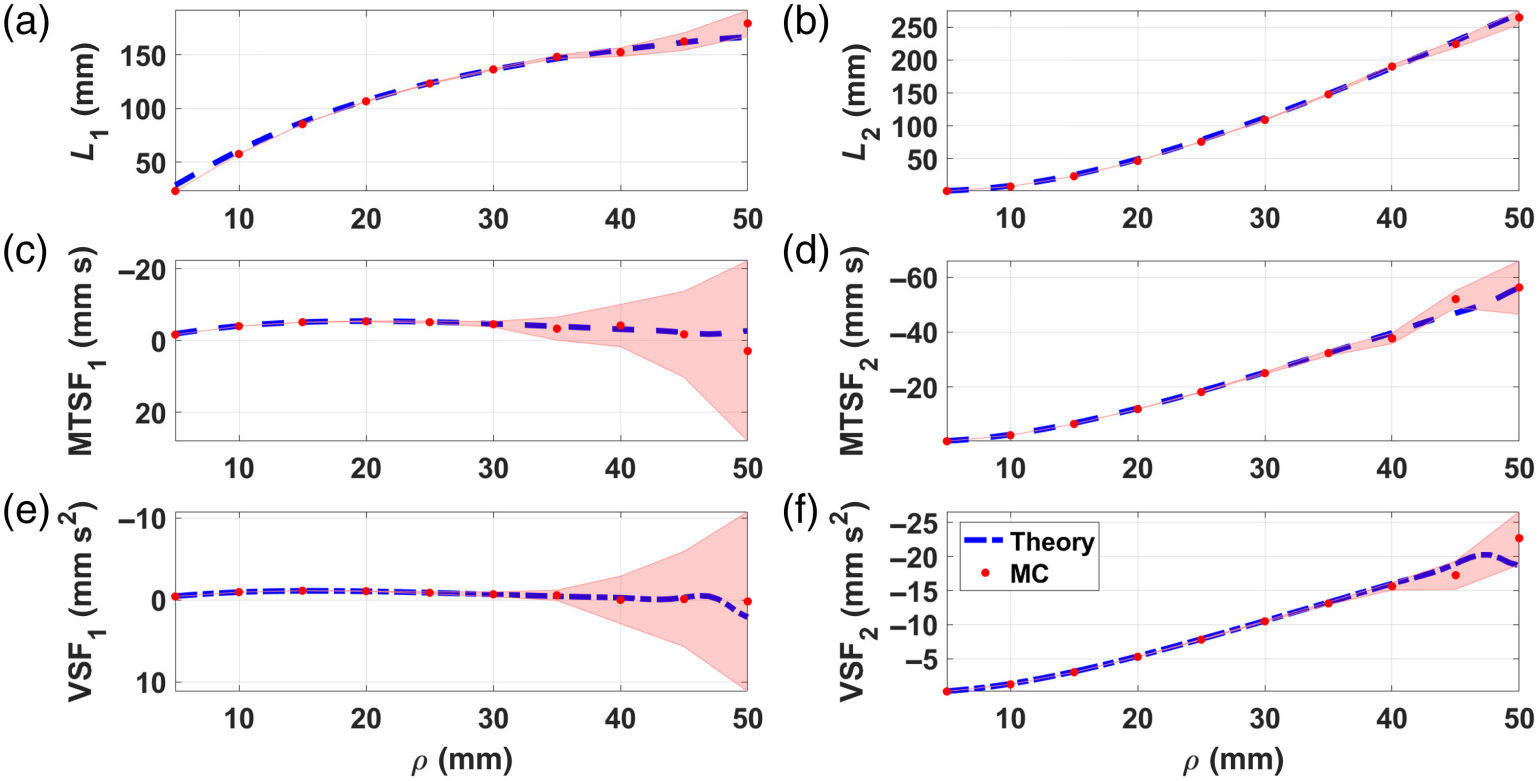
MPPLs (a) and (b), MTSFs (c) and (d), and VSFs (e) and (f)—computed for a two-layered medium, with optical and geometrical parameters given in Table 1. Blue dashed line, theory; red dots, MC. The pale red–shaded area represents the one standard deviation interval for the MC simulations.

Figure 3 shows the comparison between theory (blue dashed line) and MC simulations (red dots) for the MPPLs (a) and (b), MTSFs (c) and (d), and VSFs (e) and (f)—as a function of ρ, computed for a two-layered medium with optical and geometrical properties given in Table 1. As it can be seen, the error bands tend to increase with the source–detector distance, something expected due to the decrease in the amount of detected photons as the detector moves farther away from the source. This is particularly notorious for the MTSF and the VSF in the first layer.

**Fig. 4:**
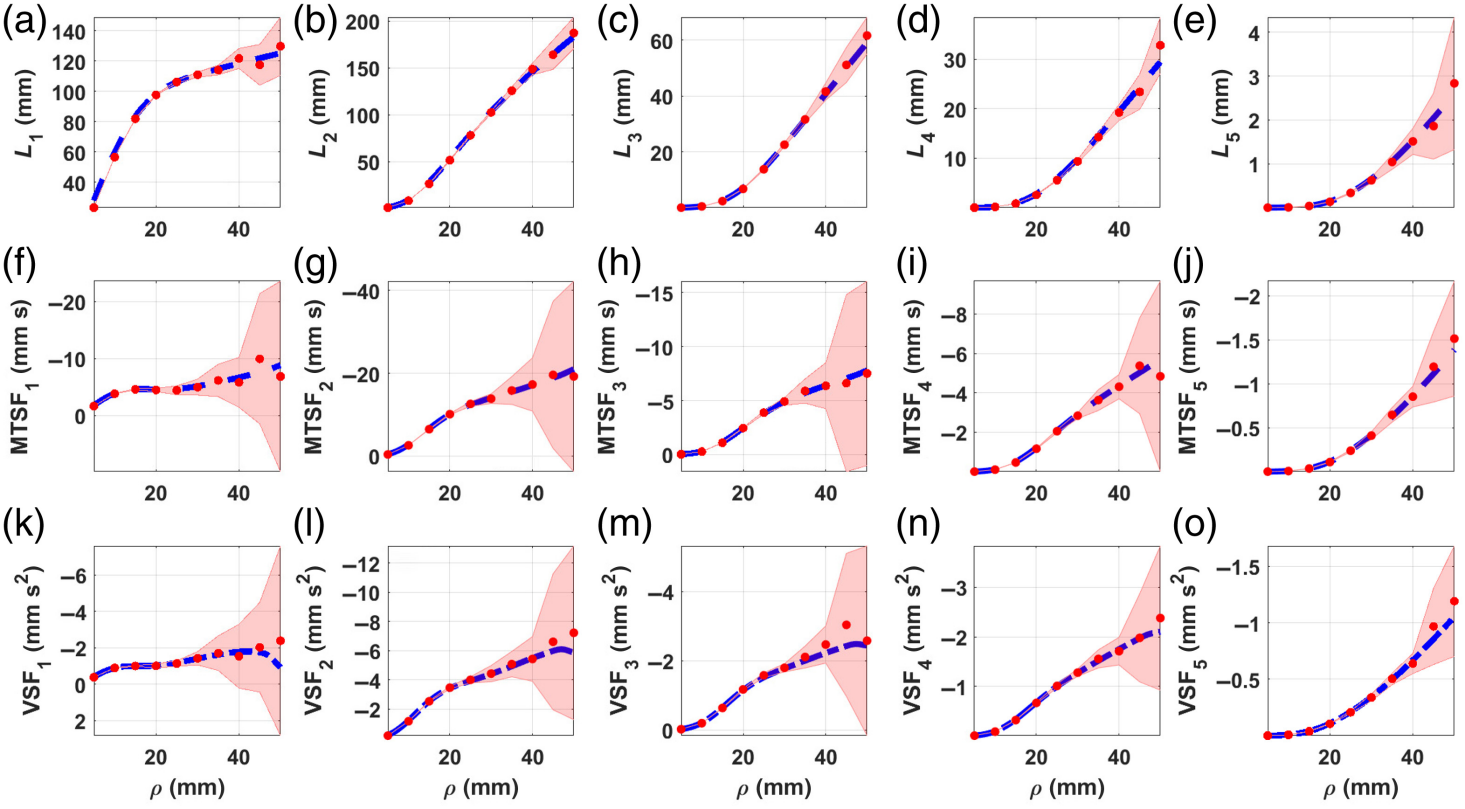
MPPLs (a)–(e), MTSFs (f)–(j), and VSFs (k)–(o)—computed for a five-layered medium, with optical and geometrical parameters given in Table 1. Blue dashed line, theory; red dots, MC. The pale red–shaded area represents the one standard deviation interval for the MC simulations.

Figure 4 shows the comparison between theory and MC for the five-layered medium. In this case, we see dramatically increasing uncertainties in the MC results for the MTSF and the VSF in all layers, especially for ρ>40  mm, which may make any comparison difficult to hold. Nevertheless, in most fNIRS applications, typical values of ρ do not exceed 40 mm, meaning that below that limit, the analytical sensitivity factors can be easily used instead of their MC counterparts without further worries.

**Fig. 5:**
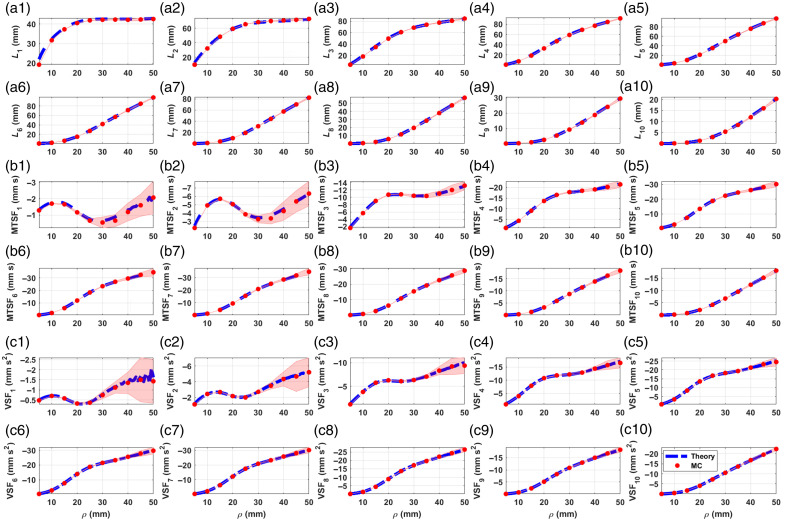
MPPLs (a1)–(a10), MTSFs (b1)–(b10), and VSFs (c1)–(c10)—computed for a 10-layered medium, with optical and geometrical parameters given in Table 1. Blue dashed line, theory; red dots, MC. The pale red–shaded area represents the one standard deviation interval for the MC simulations.

Figure 5 shows the comparison between theoretical and simulated sensitivity factors for a 10-layered medium. Surprisingly, the MC results do not show extremely large error bands, except maybe for the VSF in layers 1 and 2; the reason for this is likely the combination of optical properties set for this simulation, which favors the detection of a larger amount of photons than in the previous situation for the five-layered medium, leading to overall better statistics. In addition, in this case, it can be seen that the theory presents small oscillations for 45  mm≤ρ≤50  mm. This is due to convergence issues and can be overcome by adjusting the number of Bessel zeros used in the computation. Nevertheless, for interoptode distances up to 40 mm, the theory can be used without further concern.

**Fig. 6:**
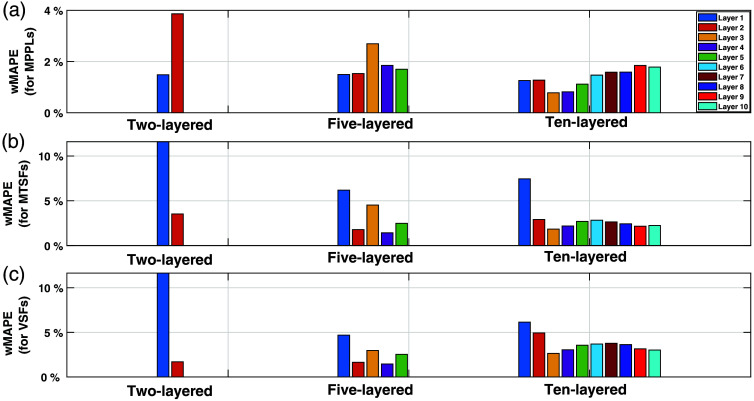
Mean absolute percentage error for (a) MPPLs, (b) MTSFs, and (c) VSFs in the 2-, 5-, and 10-layered media.

Finally, and as a metric to quantify the discrepancies between theory and MC simulations, Fig. 6 illustrates the weighted mean absolute percentage error (wMAPE) for the MPPLs, MTSFs, and VSFs in the case of the 2-, 5-, and 10-layered media. This metric is computed as^44^

$$\mathrm{wMAPE}=\frac{\overset{K}{\underset{k=1}{\sum }}|{\mathrm{SF}}_{\mathrm{MC},k}-{\mathrm{SF}}_{T,k}|}{\overset{K}{\underset{k=1}{\sum }}|{\mathrm{SF}}_{\mathrm{MC},k}|},$$ (33)

where ${\mathrm{SF}}_{\mathrm{MC}}$ and ${\mathrm{SF}}_{T}$ are the MC- and the analytically computed sensitivity factors, respectively; the index k sweeps all the source–detector distances; and K is the total amount of source–detector distances (in this study, K=10). The wMAPEs for the MPPLs remain bounded well below 10% for all layers in the three media studied here. This is also true for the other SFs in the 5- and 10-layered media, but for the two-layered media, the error in the top layer increases up to ∼12%. The reason for this is still not clear, but we speculate that it has some relation with the fact that the upper layer affects the migration of early photons much more than the lower layer, being these early photons particularly difficult to be modeled by the DA. Although the wMAPE for the ${\mathrm{MTSF}}_{1}$ and the ${\mathrm{VSF}}_{1}$ in the 5- and 10-layered media are smaller, it is always the largest, suggesting that this effect is “diluted” because of the presence of the extra layers. This issue could probably be mitigated by setting ρ>10  mm and also by discarding the early photons from DTOFs (although shorter distances and earlier detected photons could still be considered depending on the optical and geometrical properties of the layers, specially the most superficial one). Here, we would like to emphasize the label of “early photons,” which is a concept linked to time-resolved measurements; this is probably the reason why the MTSF and the VSF (quantities obtained only by time-resolved techniques) are the ones affected by this problem, but not the MPPLs (which can be obtained by CW measurements).

### 4.2 Results for Application Examples

**Fig. 7:**
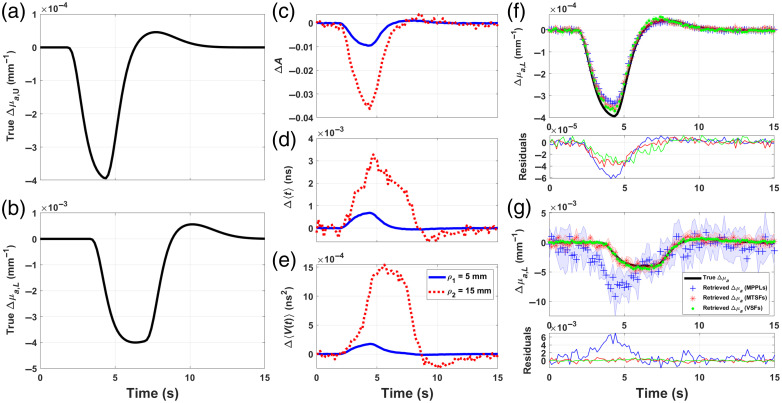
$\Delta \mu_{a}$ absorption change proposals for the first (a) and fourth (b) layers in a five-layered medium with geometrical and (initial) optical properties shown in Table 1. MC-simulated changes in attenuation (c), mean time of flight (d), and variance (e), measured with $\rho_{1}=5\;\mathrm{mm}$ and $\rho_{2}=15\;\mathrm{mm}$. Absorption changes retrieval for the upper (f) and lower (g) layers (blue cross, reconstruction with ΔA; red asterisk, reconstruction with Δ⟨t⟩; and green dot, reconstruction with ΔV); below each plot, the corresponding residuals are shown.

**Fig. 8:**
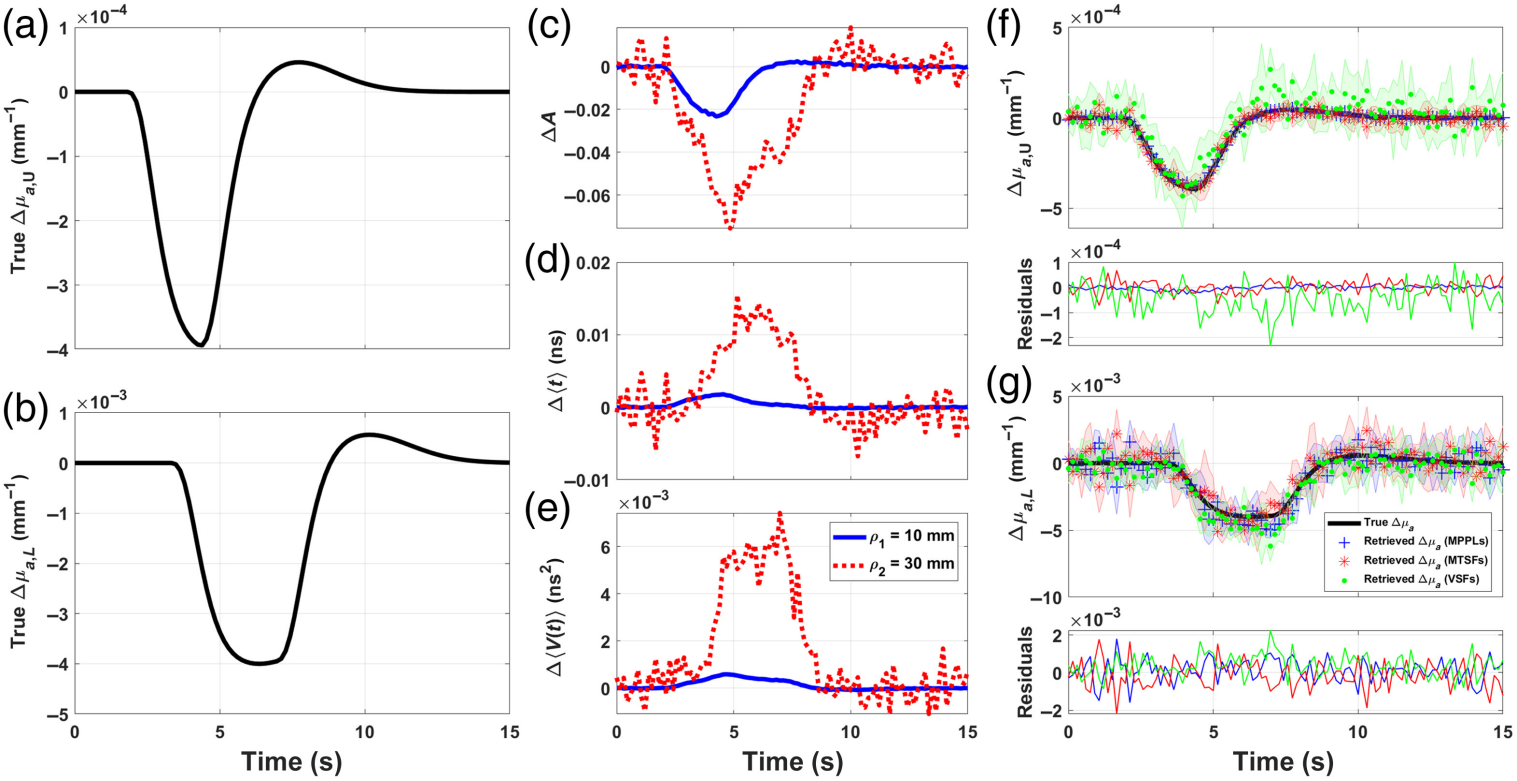
$\Delta \mu_{a}$ absorption change proposals for the first (a) and fourth (b) layers in a five-layered medium with geometrical and (initial) optical properties shown in Table 1. MC-simulated changes in attenuation (c), mean time of flight (d), and variance (e), measured with $\rho_{1}=10\;\mathrm{mm}$ and $\rho_{2}=30\;\mathrm{mm}$. Absorption changes retrieval for the upper (f) and lower (g) layers (blue crosses, reconstruction with ΔA; red asterisks, reconstruction with Δ⟨t⟩; and green dots, reconstruction with ΔV); below each plot, the corresponding residuals are shown.

Figures 7 and 8 show the proposed absorption changes in the upper and lower layers [panels (a) and (b), respectively]; the MC-generated signals for ΔA [panel (c)], Δ⟨t⟩ [panel (d)], and ΔV [panel (e)] at $(\rho_{1},\rho_{2})=(5,15)\;\mathrm{mm}$ for Fig. 7 and $(\rho_{1},\rho_{2})=(10,30)\;\mathrm{mm}$ for Fig. 8 and the reconstructions obtained using each of these moments as input data for Eq. (32). The blue, red, and green markers represent the retrievals obtained using the MPPLs, the MTSFs, and the VSFs, respectively, whereas the proposed $\Delta \mu_{a,U}$ and $\Delta \mu_{a,L}$ (black lines) are shown for comparison; the shaded area represents one standard deviation, and the thin plot below each retrieval represents the residuals obtained as the difference between the proposal and each reconstruction.

As it can be seen from Fig. 7, all the SFs perform similarly when retrieving $\Delta \mu_{a,U}$, being the MPPL retrieval the one with the largest deviation (∼15% at the peak of the change), followed by the MTSF and the VSF retrievals. In the case of the lower layer, the error associated to the MPPL retrieval is even larger (not only in terms of the reconstructed values but also in detecting the peak of the absorption change before the proposal), whereas the retrievals provided by the MTSF and the VSF are able to reproduce the proposal in a much more accurate manner, although still underestimating the true absorption change.

The previous results can be improved with the second combination of source–detector distances, as shown in Fig. 8, for which all the SFs perform similarly, producing deviations that oscillate randomly around zero for all the time interval in both layers. Although the noise distribution is high (especially for the VSF reconstruction), the average error is less than 10% for the upper layer and less than 15% for the lower layer. Noticeably, in this case, none of the reconstructions presents a shift in time with respect to the proposals.

The results from the study conducted here are in good agreement with those reported by other investigations,^9^^,^^15^^,^^16^ in the sense that SFs associated with higher-order moments are more sensitive to deeper changes. This is particularly evident for the first source–detector combination, which suggests the chosen distances are so short that even $\Delta \mu_{a,U}$ behaves like a “deep absorption change,” which explains the rather poor performance of the MPPLs when compared with the other sensitivity factors. On the other hand, the second source–detector combination performs much better, providing more accurate results for all the SFs. This does not imply by any means that $\rho_{1}$ and $\rho_{2}$ must be always set to 10 and 30 mm, respectively, because the qualitative assertion of “superficial” and “deep” changes are closely linked to the optical properties and the thickness of each layer; in any case, the more *a priori* information about the layered medium is available, the better the strategical choice of $\rho_{1}$ and $\rho_{2}$ will result, and hence, the more precise the absorption changes retrievals will be.

### 4.3 Time Cost

**Fig. 9:**
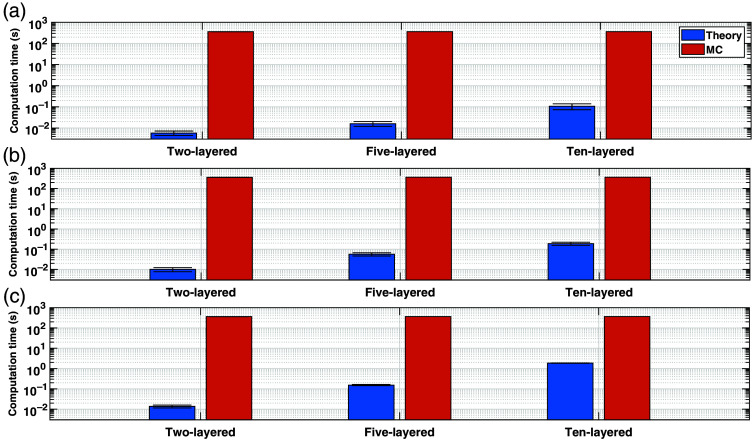
Comparison among computation times of (a) MPPLs, (b) MTSFs, and (c) VSFs by means of MC simulations (orange bars) and the analytical model (blue bars). All plots are shown in logarithmic scale.

Figure 9 shows the computation times needed to obtain the SFs for 2-, 5-, and 10-layered media using MC simulations or the analytical model introduced in this work. As expected, the time consumption associated with MC simulations is the largest (between $10^{2}$ and $10^{3}$). Regarding the analytical model, computation times vary from less than $10^{-2}$ (for the MPPLs in the two-layered case) to ∼1  s (for the VSF in the 10-layered case). This represents an improvement in time costs between 100× and 10,000×. Although 100× is a fair speedup with respect to MC, it corresponds to computation times of ∼1  s (as it was just mentioned), which signifies an important bottleneck for real-time fNIRS applications, where acquisition rates can be as high as 100 Hz.^45^ However, this result was obtained for the rather excessive scenario of a 10-layered medium; a more realistic situation is the five-layered medium, which basically represents the amount of segmented tissues in most magnetic resonance imaging (MRI) and computed tomography (CT) studies, and for which the time cost is around 0.1 s (both for the MTSF and the VSF), leading to sampling rates as high as 10 Hz, which is fairly enough in most cases.^30^ Nevertheless, it must still be taken into consideration that the code implementation used in this work is probably not the most efficient possible, so further improvements can be achieved by exploiting good programming practices and/or using other, more “low-level” languages such as C/C++.

## 5 Conclusion

In this work, we introduced a method for computing the so-called sensitivity factors of DTOFs, i.e., mean partial pathlengths (MPPLs), MTSF, and VSF, in an analytical fashion. We based our model on the TD diffuse reflectance for layered turbid media under the diffusion approximation (DA) and compared our results with Monte Carlo simulations run in 2-, 5-, and 10-layered media, which resulted in a very good agreement in almost all cases, except for the mean time and the variance sensitivity factors in the top layer when considering a two-layered medium; the reason for these discrepancies must be further investigated, but we hypothesize that it has some relation with the lack of precision of the DA in modeling early photons in those particular cases, as discussed by the end of Sec. 4.1; in the remaining cases, we would like to stress once again the excellent results provided by the theory, additionally with increased spatial resolution, without the typical stochastic noise from MC simulations and without special hardware capabilities such as GPUs. The codes that reproduce the analytical results shown in this paper can be found in Ref. 25.

Moreover, we introduced examples of absorption change reconstructions in the case of a five-layered medium mimicking the tissues present in the human head. We confirmed previous findings stating that the sensitivity to depth of the reconstruction method is closely associated with the order of the statistical moment used for the retrieval. This means that a correct choice of a source–detector pair of distances will optimize the outcome of the measurements; however, knowing this optimal combination of optodes is not always possible because this depends on the optical and geometrical characteristics of the studied medium, so as much *a priori* information as possible must be used whenever available to reduce uncertainties.

Although the results reported in this article are based on the diffusion approximation, this analytical method can be extended to more general cases, such as higher-order approximations to the radiative transfer equation. ^46^^,^^47^ Besides, other geometries (slabs, spheres), other heterogeneities (such as inclusions), and other measurement configurations (such as transmittance) and techniques (frequency-domain) can be used. The only requisite is having analytical expressions for the signal to be derived with respect to the different absorption coefficients.

It must be noted that the results and the conclusions discussed in this work are strongly based on the hypothesis that hemodynamic changes only impact the absorption coefficient, whereas variations in the reduced scattering coefficient are assumed negligible. Although this is in general true when fNIRS is applied to brain hemodynamics^1^^,^^7^^,^^9^^,^^29^^,^^30^^,^^48^ (where changes occur over time), this might not be the case for other applications (such as optical mammography), where spatial changes associated to tissue structure may have a stronger impact on $\mu_{s}^{\prime }$.^49^^,^^50^ Hence, a possible extension to this work could include deriving the corresponding SFs that account for changes in the reduced scattering coefficient as well.

## Supplementary Material



## Data Availability

The code used to generate the results and figures is available in a GitHub repository: https://github.com/DemianVera/SensitivityFactors
