# Supplementary material for: Analytical sensitivity factors from distributions of time of flight of photons for near-infrared spectroscopy studies in multilayered turbid media
Source: J Biomed Opt. 2025 Jan 22;30(1):015002. doi: 10.1117/1.JBO.30.1.015002 (PMC11753179; doi:10.1117/1.JBO.30.1.015002)
Supplement: Supplementary file 1 [file JBO_030_015002_SD001.pdf]

Analytical sensitivity factors from distributions of time of flight of photons for Near Infrared Spectroscopy studies in multilayered turbid media - Supplementary material

Héctor A. García[a,b,*], **Demián A. Vera**[b], **Nicolás A. Carbone**[b], **María V. Waks-Serra**[b], **Juan A. Pomarico**[b]

[a]Department of Medical Physics, School of Medicine and Public Health, UW-Madison, 1111 Highland Ave., 53705, Madison, USA

[b]CIFICEN (UNCPBA - CICPBA - CONICET), Pinto 399, B7000GHG, Tandil, Buenos Aires, Argentina

1 Supplementary material

This supplementary material is intended to provide a deeper insight into the calculation of the sensitivity factors (SFs) of distributions of time of flight (DTOFs) of photons for the particular case of a semiinfinite multilayared turbid medium.

1.1 Mean partial pathlengths (MPPLs)

Although the general relations for the MPPLs have been derived in previous works,[?] here we repeat the details since they will become useful for deriving the quantities associated to the remaining SFs. We start with Eq. (9) of the main manuscript:

$$L_j = -\frac{1}{R}\frac{\partial R}{\partial \alpha_j}\frac{\partial \alpha_j}{\mu_{a,j}}, \tag{1}$$

with R being the diffuse reflectance, that depends on a Green's function for the first layer as:

$$R = \frac{1}{4\pi^2 A R_{EB}} \sum_{n=1}^{\infty} \frac{J_0(s_n \rho)}{J_1^2(s_n R_{EB})} G_1(z=0,\omega,s_n), \tag{2}$$

where the Green's function —Eq. (2) of the manuscript— can be re-expressed as:

$$G_1(\boldsymbol{\alpha}) = \mathcal{A}(\alpha_1) + \mathcal{B}(\alpha_1) \times \mathcal{C}(\alpha_1, \alpha_2, ..., \alpha_N). \tag{3}$$

From now on, we focus our attention on the derivatives of Eq. (1). For the sake of brevity, the derivatives with respect to α_i will be denoted as:

$$\frac{\partial R}{\partial \alpha_j} = \partial_j R, \qquad \frac{\partial^2 R}{\partial \alpha_m \partial \alpha_j} = \partial_{jm} R, \qquad \frac{\partial^3 R}{\partial \alpha_n \partial \alpha_m \partial \alpha_j} = \partial_{jmn} R. \tag{4}$$

According to expression (2), the first order derivative of the reflectance R yields:

$$\partial_j R = \frac{1}{4\pi^2 A R_{EB}} \sum_{n=1}^{\infty} \frac{J_0(s_n \rho)}{J_1^2(s_n R_{EB})} \partial_j G_1(z = 0, \omega, s_n), \tag{5}$$

Now, following expression (3), $\partial_j G_1$ can be obtained as:

$$\partial_j G_1 = \partial_j \mathcal{A} \cdot \delta_{1,j} + \partial_j \mathcal{B} \cdot \mathcal{C} \cdot \delta_{1,j} + \partial_j \mathcal{C} \cdot \mathcal{B}, \tag{6}$$

being $\delta_{i,j}$ the Kronecker delta. For the first term, we obtain:

$$\partial_j \mathcal{A} \cdot \delta_{1,j} = \partial_1 \mathcal{A} = \frac{e^{-\alpha_1(z_0+2z_b)} \left[(z_0 + 2z_b)\alpha_1 + 1\right] - e^{-\alpha_1 z_0}(\alpha_1 z_0 + 1)}{2D_1 \alpha_1^2}. \tag{7}$$

For the second term we have to compute $\partial_j B \cdot \delta_{1,j} = \partial_1 B$. First, we will write $\mathcal{B}$ as:

$$\mathcal{B} = \frac{\sinh\left[\alpha_1(z_0 + z_b)\right] \sinh\left[\alpha_1 z_b\right]}{D_1 \alpha_1 e^{d_1 + z_b}} = \frac{S_0 S_1}{S_2}, \tag{8}$$

where it is clear that:

$$S_0 = \sinh\left[\alpha_1(z_0 + z_b)\right]. \tag{9}$$

$$S_1 = \sinh\left[\alpha_1 z_b\right]. \tag{10}$$

$$S_2 = D_1\alpha_1 e^{d_1 + z_b}. \tag{11}$$

Thus, $\partial_1\mathcal{B}$ takes the form:

$$\partial_1\mathcal{B} = \frac{\partial_1 S_0 \cdot S_1 + \partial_1 S_0 \cdot S_1 - \mathcal{B}\cdot\partial_1 S_2}{S_2}, \tag{12}$$

with:

$$\partial_1 S_0 = (z_0 + z_b)\cosh\left[\alpha_1(z_0 + z_b)\right]. \tag{13}$$

$$\partial_1 S_1 = z_b \cosh\left[\alpha_1 z_b\right]. \tag{14}$$

$$\partial_1 S_2 = D_1 e^{d_1 + z_b}\left[1 + \alpha_1(d_1 + z_b)\right]. \tag{15}$$

We continue with the first order derivative of $\mathcal{C} = \delta/\Delta$.

$$\partial_j\mathcal{C} = \partial_j\left(\frac{\delta}{\Delta}\right) = \left[\partial_j\delta\cdot\Delta - \delta\cdot\partial_j\Delta\right]\Delta^{-2}. \tag{16}$$

Here,

$$\delta = D_1\alpha_1 n_1^2\beta_3 - D_2\alpha_2 n_2^2\gamma_3, \tag{17}$$

and

$$\Delta = D_1\alpha_1 n_1^2\beta_3 \cosh\left[\alpha_1(d_1+z_b)\right] + D_2\alpha_2 n_2^2\gamma_3 \sinh\left[\alpha_1(d_1+z_b)\right], \tag{18}$$

where β_3 and γ_3 can be computed with the help of Eqs. (3) and (4) in the main manuscript. Hence,

$$\partial_j\delta = D_1 n_1^2\left(\delta_{1,j}\beta_3 + \alpha_1\partial_j\beta_3\right) - D_2 n_2^2\left(\delta_{2,j}\gamma_3 + \alpha_2\partial_j\gamma_3\right). \tag{19}$$

The first order derivative of Δ can be stated as:

$$\begin{gathered}\partial_j\Delta = \left[D_1 n_1^2\left(\delta_{1,j}\beta_3 + \alpha_1\partial_j\beta_3\right) + D_2 n_2^2\alpha_2\gamma_3(d_1+z_b)\delta_{1,j}\right]\cosh\left[\alpha_1(d_1+z_b)\right] + \\ \left[D_2 n_2^2\left(\delta_{2,j}\gamma_3 + \alpha_2\partial_j\gamma_3\right) + D_1 n_1^2\alpha_1\beta_3(d_1+z_b)\delta_{1,j}\right]\sinh\left[\alpha_1(d_1+z_b)\right].\end{gathered} \tag{20}$$

Before computing the quantities $\partial_j\beta_3$ and $\partial_j\gamma_3$, we show explicitly the recursive relations for β_3 and γ_3 that can be expressed as:

$$\beta_{k-1} = D_{k-2}n_{k-2}^2\cosh\left(\alpha_{k-2}d_{k-2}\right)\beta_k + D_{k-1}n_{k-1}^2\sinh\left(\alpha_{k-2}d_{k-2}\right)\gamma_k. \tag{21}$$

$$\gamma_{k-1} = D_{k-2}n_{k-2}^2\sinh\left(\alpha_{k-2}d_{k-2}\right)\beta_k + D_{k-1}n_{k-1}^2\cosh\left(\alpha_{k-2}d_{k-2}\right)\gamma_k. \tag{22}$$

The initial values for β_{k-1} and γ_{k-1} are:

$$\beta_N = D_{N-1}n_{N-1}^2\cosh\left(\alpha_{N-1}d_{N-1}\right) + D_N n_N^2\sinh\left(\alpha_{N-1}d_{N-1}\right), \tag{23}$$

$$\gamma_N = D_{N-1}n_{N-1}^2\sinh\left(\alpha_{N-1}d_{N-1}\right) + D_N n_N^2\cosh\left(\alpha_{N-1}d_{N-1}\right), \tag{24}$$

where N is the number of layers of the medium. Now we are ready to compute $\partial_j \beta_3$ and $\partial_j \gamma_3$:

$$\begin{aligned}\partial_j \beta_{k-1} = &\left[D_{k-2} n_{k-2}^2 \left(\delta_{k-2,j} \beta_k + \alpha_{k-2} \partial_j \beta_k\right) + D_{k-1} n_{k-1}^2 \delta_{k-2,j} d_{k-2} \alpha_{k-1} \gamma_k\right] \cosh\left(\alpha_{k-2} d_{k-2}\right) + \\ &\left[D_{k-1} n_{k-1}^2 \left(\delta_{k-1,j} \gamma_k + \alpha_{k-1} \partial_j \gamma_k\right) + D_{k-2} n_{k-2}^2 d_{k-2} \delta_{k-2,j} \alpha_{k-2} \beta_k\right] \sinh\left(\alpha_{k-2} d_{k-2}\right)\end{aligned} \tag{25}$$

$$\begin{aligned}\partial_j \gamma_{k-1} = &\left[D_{k-2} n_{k-2}^2 \left(\delta_{k-2,j} \beta_k + \alpha_{k-2} \partial_j \beta_k\right) + D_{k-1} n_{k-1}^2 \delta_{k-2,j} d_{k-2} \alpha_{k-1} \gamma_k\right] \sinh\left(\alpha_{k-2} d_{k-2}\right) + \\ &\left[D_{k-1} n_{k-1}^2 \left(\delta_{k-1,j} \gamma_k + \alpha_{k-1} \partial_j \gamma_k\right) + D_{k-2} n_{k-2}^2 d_{k-2} \delta_{k-2,j} \alpha_{k-2} \beta_k\right] \cosh\left(\alpha_{k-2} d_{k-2}\right).\end{aligned} \tag{26}$$

And the derivatives of the starting values read:

$$\begin{aligned}\partial_j \beta_N = &\left(D_{N-1} n_{N-1}^2 + D_N n_N^2 \alpha_N d_{N-1}\right) \delta_{N-1,j} \cosh(\alpha_{N-1} d_{N-1}) + \\ &\left(D_{N-1} n_{N-1}^2 \alpha_{N-1} d_{N-1} \delta_{N-1,j} + D_N n_N^2 \delta_{N,j}\right) \sinh(\alpha_{N-1} d_{N-1}).\end{aligned} \tag{27}$$

$$\begin{aligned}\partial_j \gamma_N = &\left(D_{N-1} n_{N-1}^2 + D_N n_N^2 \alpha_N d_{N-1}\right) \delta_{N-1,j} \sinh(\alpha_{N-1} d_{N-1}) + \\ &\left(D_{N-1} n_{N-1}^2 \alpha_{N-1} d_{N-1} \delta_{N-1,j} + D_N n_N^2 \delta_{N,j}\right) \cosh(\alpha_{N-1} d_{N-1}).\end{aligned} \tag{28}$$

Here, it must be noted that:

$$\delta_{N-1,j} = 1, \quad N-1 = j. \tag{29}$$

$$\delta_{N-1,j} = 0, \quad N-2 \geq j. \tag{30}$$

$$\delta_{N,j} = 1, \qquad N = j. \tag{31}$$

We point out that for a two-layered medium, $\beta_3 = \gamma_3 = 1$.

As a last remark, we note that:

$$\frac{\partial \alpha_i}{\partial \mu_{a,j}} = \frac{1}{2D_i\alpha_i}\delta_{i,j}. \tag{32}$$

1.2 Mean time sensitivity factors (MTSFs)

We move now to the mean time sensitivity factor. The second order derivative of $R(\rho)$ from Eq. (16) in the main manuscript can be expressed as:

$$\frac{\partial^2 R}{\partial \mu_{a,m}\mu_{a,j}} = \frac{\partial^2 \alpha_j}{\partial \mu_{a,m}\partial \mu_{a,j}}\partial_j R + \frac{\partial \alpha_j}{\partial \mu_{a,j}}\frac{\partial \alpha_m}{\partial \mu_{a,m}}\partial_{jm} R. \tag{33}$$

The second order derivative in the last term of expression (33) is the only non-trivial quantity that has not been computed yet. To obtain it, we start by noting that the second order derivative of R, according to expression (3), is:

$$\begin{aligned}\partial_{jm} G_1 =& \partial_{jm}\mathcal{A}\delta_{1,j}\delta_{1,m} + \mathcal{C}\cdot\partial_{jm}\mathcal{B}\delta_{1,j}\delta_{1,m} + \\ & \partial_j\mathcal{B}\delta_{1,j}\cdot\partial_m\mathcal{C} + \partial_m\mathcal{B}\delta_{1,m}\cdot\partial_j\mathcal{C} + \mathcal{B}\cdot\partial_{jm}\mathcal{C}.\end{aligned} \tag{34}$$

The first order derivatives of $\mathcal{B}$ and $\mathcal{C}$ have been computed above; we focus now on computing $\partial_{jm}\mathcal{A}$, $\partial_{jm}\mathcal{B}$ and $\partial_{jm}\mathcal{C}$. We have, then:

$$\begin{aligned}&\partial_{jm}\mathcal{A}\delta_{1,j}\delta_{1,m}\delta_{1,j} = \partial_{11}\mathcal{A} = \\ &\qquad \frac{e^{-\alpha_1 z_0}\left[z_0^2\alpha_1^2 + 2(z_0\alpha_1 + 1)\right] - e^{-\alpha_1(z_0+2z_b)}\{(z_0+2z_b)^2\alpha_1^2 + [(z_0+2z_b)\alpha_1 + 1]\}}{2D_1\alpha_1^3}.\end{aligned} \tag{35}$$

The second order derivative of $\mathcal{B}$ is more complicated; expressing again $\mathcal{B}$ as: $\mathcal{B} = S_0S_1/S_2$, we find:

$$\begin{aligned}\partial_{11}\mathcal{B} = S_2^{-1}\Big\{&S_1\cdot\partial_{11}S_0 + S_0\cdot\partial_{11}S_1 + 2\partial_1S_0\partial_1S_1 - 2S_2^{-1}\partial_1D\left(S_1\partial_1S_0 + S_0\partial_1S_1\right) + \\ &S_2^{-2}S_0S_1\left[2S_2^{-1}\left(\partial_1S_2\right)^2 - \partial_{11}S_2\right]\Big\},\end{aligned} \tag{36}$$

where:

$$\partial_{11}S_0 = (z0 + z_b)^2\sinh\left[\alpha_1(z_0+z_b)\right] = (z_0+z_b)^2 S_0. \tag{37}$$

$$\partial_{11}S_1 = z_b^2\sinh\left(\alpha_1 z_b\right) = z_b^2 S_1. \tag{38}$$

$$\partial_{11}S_2 = D_1(d_1+z_b)e^{\alpha_1(d_1+z_b)}\left[2 + \alpha_1(d_1+z_b)\right]. \tag{39}$$

The second order derivatives of $\mathcal{C}$ are:

$$\begin{aligned}\partial_{jm}\mathcal{C} = \partial_m\Big\{\left[\partial_j\delta\cdot\Delta - \delta\cdot\partial_j\Delta\right]\Delta^{-2}\Big\} = &\left[\partial_{jm}\delta\cdot\Delta + \partial_j\delta\cdot\partial_m\Delta - \left(\partial_m\delta\cdot\partial_j\Delta + \delta\cdot\partial_{jm}\Delta\right)\right]\cdot\Delta^{-2} - \\ &2\left[\partial_j\delta\cdot\Delta - \delta\cdot\partial_j\Delta\right]\Delta^{-3}\cdot\partial_m\Delta.\end{aligned} \tag{40}$$

The term $\partial_{jm}\delta$ becomes:

$$\begin{aligned}\partial_{jm}\delta =& D_1 n_1^2 \left(\delta_{1,j}\partial_m\beta_3 + \delta_{1,m}\partial_j\beta_3 + \alpha_1\partial_{jm}\beta_3\right) - \\ & D_2 n_2^2 \left(\delta_{2,j}\partial_m\gamma_3 + \delta_{2,m}\partial_j\gamma_3 + \alpha_2\partial_{jm}\gamma_3\right).\end{aligned} \tag{41}$$

The term $\partial_{jm}\Delta$ is:

$$\begin{aligned}\partial_{jm}\Delta =& \Big\{ D_1 n_1^2 \left(\delta_{1,j}\partial_m\beta_3\delta_{1,m}\partial_j\beta_3 + \alpha_1\partial_{jm}\beta_3\right) + D_2 n_2^2 \left(\delta_{2,m}\gamma_3 + \alpha_2\partial_j\gamma_3\right)(d_1 + z_b)\delta_{1,j} + \\ & \left[D_2 n_2^2 \left(\delta_{2,j}\gamma_3 + \alpha_2\partial_j\gamma_3\right) + D_1 n_1^2\alpha_1\beta_3(d_1 + z_b)\delta_{1,j}\right]\delta_{1,m}(d_1 + z_b)\Big\} \cosh\left[\alpha_1(d_1 + z_b)\right] + \\ & \Big\{ D_2 n_2^2 \left(\delta_{2,j}\partial_m\gamma_3\delta_{2,m}\partial_j\gamma_3 + \alpha_2\partial_{jm}\gamma_3\right) + D_1 n_1^2 \left(\delta_{1,m}\beta_3 + \alpha_1\partial_j\beta_3\right)(d_1 + z_b)\delta_{1,j} + \\ & \left[D_1 n_1^2 \left(\delta_{1,j}\beta_3 + \alpha_1\partial_j\beta_3\right) + D_2 n_2^2\alpha_2\gamma_3(d_1 + z_b)\delta_{1,j}\right]\delta_{1,j}(d_1 + z_b)\Big\} \sinh\left[\alpha_1(d_1 + z_b)\right].\end{aligned} \tag{42}$$

Now, the quantities $\partial_{jm}\beta_3$ and $\partial_{jm}\gamma_3$ can be obtained in a fully general manner; to this end, we note that $\partial_j\beta_{k-1}$ can be expressed as:

$$\partial_j\beta_{k-1} = A\cosh\left(\alpha_{k-2}d_{k-2}\right) + B\sinh\left(\alpha_{k-2}d_{k-2}\right). \tag{43}$$

$$\partial_j\gamma_{k-1} = A\sinh\left(\alpha_{k-2}d_{k-2}\right) + B\cosh\left(\alpha_{k-2}d_{k-2}\right). \tag{44}$$

Here, A and B are:

$$A = D_{k-2}n_{k-2}^2\left(\delta_{k-2,j}\beta_k + \alpha_{k-2}\partial_j\beta_k\right) + D_{k-1}n_{k-1}^2\delta_{k-2,j}d_{k-2}\alpha_{k-1}\gamma_k. \tag{45}$$

$$B = D_{k-1}n_{k-1}^2\left(\delta_{k-1,j}\gamma_k + \alpha_{k-1}\partial_j\gamma_k\right) + D_{k-2}n_{k-2}^2\delta_{k-2,j}d_{k-2}\alpha_{k-2}\beta_k. \tag{46}$$

So $\partial_{jm}\beta_3$ is:

$$\partial_{jm}\beta_3 = (\partial_m A + B\delta_{k-2,m}d_{k-2})\cosh(\alpha_{k-2}d_{k-2}) + (\partial_m B + A\delta_{k-2,m}d_{k-2})\sinh(\alpha_{k-2}d_{k-2}). \tag{47}$$

With:

$$\partial_m A = D_{k-2}n_{k-2}^2\left(\delta_{k-2,m}\partial_j\beta_k + \delta_{k-2,j}\partial_m\beta_k + \alpha_{k-2}\partial_{jm}\beta_k\right) + D_{k-1}n_{k-1}^2\delta_{k-2,j}d_{k-2}\left(\delta_{k-1,m}\gamma_k + \alpha_{k-1}\partial_m\gamma_k\right). \tag{48}$$

$$\partial_m B = D_{k-1}n_{k-1}^2\left(\delta_{k-1,m}\partial_j\gamma_k + \delta_{k-1,j}\partial_m\gamma_k + \alpha_{k-1}\partial_{jm}\gamma_k\right) + D_{k-2}n_{k-2}^2\delta_{k-2,j}d_{k-2}\left(\delta_{k-2,m}\beta_k + \alpha_{k-2}\partial_m\beta_k\right). \tag{49}$$

And *mutatis mutandis* for $\partial_{jm}\gamma$.

The derivatives of the starting values, $\partial_{jm}\beta_N$, $\partial_{jm}\gamma_N$ are:

$$\partial_{jm}\beta_N = \left[D_N n_N^2 d_{N-1}\left(\delta_{N,m}\delta_{N-1,j} + \delta_{N-1,m}\delta_{N,j}\right) + D_{N-1}n_{N-1}^2\alpha_{N-1}d_{N-1}^2\delta_{N-1,j}\delta_{N-1,m}\right]\cosh(\alpha_{N-1}d_{N-1}) + \left[2D_{N-1}n_{N-1}^2 d_{N-1}\delta_{N-1,j}\delta_{N-1,m} + D_N n_N^2\alpha_N d_{N-1}^2\delta_{N-1,m}\delta_{N-1,j}\right]\sinh(\alpha_{N-1}d_{N-1}). \tag{50}$$

$$\partial_{jm}\gamma_N = \left[D_N n_N^2 d_{N-1}\left(\delta_{N,m}\delta_{N-1,j} + \delta_{N-1,m}\delta_{N,j}\right) + D_{N-1}n_{N-1}^2\alpha_{N-1}d_{N-1}^2\delta_{N-1,j}\delta_{N-1,m}\right]\sinh(\alpha_{N-1}d_{N-1}) + \left[2D_{N-1}n_{N-1}^2 d_{N-1}\delta_{N-1,j}\delta_{N-1,m} + D_N n_N^2\alpha_N d_{N-1}^2\delta_{N-1,m}\delta_{N-1,j}\right]\cosh(\alpha_{N-1}d_{N-1}). \tag{51}$$

Finally, the last derivative needed to fully calculate expression (33) is:

$$\frac{\partial^2 \alpha_k}{\partial \mu_{a,m} \partial \mu_{a,j}} = -\frac{1}{4 D_k^2 \alpha_k^3} \delta_{j,k} \delta_{m,k}. \tag{52}$$

1.3 Variance sensitivity factors (VSFs)

The third order derivatives of the Green's function G_1 can be written as:

$$\begin{aligned}\partial_{jmn} G_1 =& \partial_{jmn} \mathcal{A} \cdot \delta_{1,j} \delta_{1,m} \delta_{1,n} + \partial_{jmn} \mathcal{B} \cdot \delta_{1,j} \delta_{1,m} \delta_{1,n} \cdot \mathcal{C} + \partial_{jm} \mathcal{B} \cdot \delta_{1,j} \delta_{1,m} \cdot \partial_n \mathcal{C} + \\ & \partial_{mn} \mathcal{B} \cdot \delta_{1,m} \delta_{1,n} \cdot \partial_j \mathcal{C} + \partial_{jn} \mathcal{B} \cdot \delta_{1,j} \delta_{1,n} \cdot \partial_m \mathcal{C} + \partial_{jm} \mathcal{C} \cdot \partial_n \mathcal{B} \cdot \delta_{1,n} + \\ & \partial_{jn} \mathcal{C} \cdot \partial_m \mathcal{B} \cdot \delta_{1,m} + \partial_{mn} \mathcal{C} \cdot \partial_j \mathcal{B} \cdot \delta_{1,j} + \partial_{jmn} \mathcal{C} \cdot \mathcal{B}.\end{aligned} \tag{53}$$

Now, the derivatives $\partial_{jmn} \mathcal{A} \cdot \delta_{1,j} \delta_{1,m} \delta_{1,n}$, $\partial_{jmn} \mathcal{B} \cdot \delta_{1,j} \delta_{1,m} \delta_{1,n}$ and $\partial_{jmn} \mathcal{C}$ are needed. For the first one, we have:

$$\partial_{111} \mathcal{A} = \frac{\left[x_1^3 + 3x_1^2 + 6(x_1 + 1)\right] e^{-x_1} - \left[x_2^3 + 3x_2^2 + 6(x_2 + 1)\right] e^{-x_2}}{2 D_1 \alpha_1^4}, \tag{54}$$

where $x_1 = \alpha_1 \left(z_0 + 2z_b\right)$ and $x_2 = \alpha_1 z_0$.

The second term of Eq. (53) can be found by deriving it with respect to α_1. For the sake of brevity, we will simplify the notation by replacing ∂_1 with primes as super-indices since in this case there is no possible confusion:

$$\mathcal{B}''' = -S_2^{-2}S_2'\left\{S_1S_0' + S_0S_1'' + 2S_0'S_1' - 2S_2^{-1}S_2'\left(S_1S_0' + S_0S_1'\right) + S_2^{-2}S_0S_1\left[2S_2^{-1}\left(S_2'\right)^2 - S_2''\right]\right\} +$$
$$\left\{S_0S_1''' + S_1S_0''' + 3\left(S_0''S_1' + S_1''S_0\right) + 2S_2^{-2}S_2'\left[S_2'(S_1S_0' + S_0S_1')\right] - 2S_2^{-1} \times \left[S_2''(S_1S_0' + S_0S_1') - S_2'\right.\right.$$
$$\left.(2S_1'S_0' + S_1S_0'' + S_0S_1'')\right] - 2S_2^{-3}S_2'S_0S_1\left(2S_2^{-1}S_2'^2 - S_2''\right) + S_2^{-2}(S_0'S_1 + S_0S_1')\left(2S_2^{-1}S_2'^2 - S_2''\right)$$
$$\left.S_2^{-2}S_0S_1\left[-2S_2^{-2}S_2'S_2'^2 + 4S_2^{-1}S_2'S_2'' - S_2'''\right]\right\}. \tag{55}$$

72 Here, S_0'', S_1'' and S_2'' are the same as in the expressions (37) to (39), and:

$$S_0''' = (z_0 + z_b)^3 \cosh\left[\alpha_1(z_0 + z_b)\right]. \tag{56}$$

$$S_1''' = z_b^3 \cosh\left[\alpha_1(z_b)\right]. \tag{57}$$

$$S_2''' = D_1(d_1 + z_b)^2 e^{\alpha_1(d_1+z_b)}\left[3 + \alpha_1(d_1 + z_b)\right]. \tag{58}$$

73 The remaining derivative, i.e. $\partial_{jmn}\mathcal{C}$, can be written as follows:

$$\begin{aligned}\partial_{jmn}\mathcal{C} = &[\partial_{mn}\Delta \cdot \partial_j\delta + \partial_m\Delta \cdot \partial_{jn}\delta + \partial_n\Delta \cdot \partial_{jm}\delta + \Delta \cdot \partial_{jmn}\delta - \\ &(\partial_{mn}\delta \cdot \partial_j\Delta + \partial_m\delta \cdot \partial_{jn}\Delta + \partial_{jmn}\Delta \cdot \delta + \partial_{jm}\Delta \cdot \partial_n\delta)]\,\Delta^{-2} - \\ &2\Delta^{-3}\partial_p\Delta\left[\partial_{jm}\delta \cdot \Delta + \partial_j\delta \cdot \partial_m\Delta - (\partial_m\delta \cdot \partial_j\Delta + \delta \cdot \partial_{jm}\Delta)\right] + \\ &2\left[\Delta^4\partial_p\Delta\partial_k\Delta - \Delta^{-3}\partial_{mn}\Delta\right](\partial_k\delta \cdot \partial_m\Delta + \partial_{km}\Delta \cdot \delta) - \\ &2\Delta^{-3}\partial_k\Delta \cdot \partial_n\left(\partial_k\delta \cdot \partial_m\Delta + \partial_{km}\Delta \cdot \delta\right).\end{aligned} \tag{59}$$

74 Where $\partial_{jmn}\delta$ is:

$$\partial_{jmn}\delta = D_1 n_1^2 \left(\delta_{1,m} \cdot \partial_{jn}\beta_3 + \delta_{1,n} \cdot \partial_{jm}\beta_3 + \delta_{1,j} \cdot \partial_{mn}\beta_3 + \alpha_1 \partial_{jmn}\beta_3\right) - D_2 n_2^2 \left(\delta_{2,m} \cdot \partial_{jn}\gamma_3 + \delta_{2,n} \cdot \partial_{jm}\gamma_3 + \delta_{2,j} \cdot \partial_{mn}\gamma_3 + \alpha_2 \partial_{jmn}\gamma_3\right) \tag{60}$$

Here, the second order derivatives of β_3 and γ_3 are the ones found in the previous Section, and the third order derivatives $\partial_{jmn}\beta_3$ and $\partial_{jmn}\gamma_3$ are to be found. To do so, once again we make use of the recurrence relations β_{k-1} and γ_{k-1}. First, we note that, as shown in the previous Section, the second order derivatives of β_{k-1} and γ_{k-1} have the form:

$$\partial_{jm}\beta_{k-1} = \left[\Lambda + d_{k-2}\delta_{k-2,m} \cdot B\right] \cosh\left(\alpha_{k-2}d_{k-2}\right) + \left[\Gamma + \delta_{k-2,m}d_{k-2} \cdot A\right] \sinh\left(\alpha_{k-2}d_{k-2}\right). \tag{61}$$

$$\partial_{jm}\gamma_{k-1} = \left[\Lambda + d_{k-2}\delta_{k-2,m} \cdot B\right] \sinh\left(\alpha_{k-2}d_{k-2}\right) + \left[\Gamma + \delta_{k-2,m}d_{k-2} \cdot A\right] \cosh\left(\alpha_{k-2}d_{k-2}\right), \tag{62}$$

where $\Lambda = \partial_m A$ and $\Gamma = \partial_m B$. With this in mind, the third order derivatives of β_{k-1} and γ_{k-1} become:

$$\partial_{jmn}\beta_{k-1} = \left[\partial_n\Lambda + d_{k-2} \cdot \delta_{k-2,m} \cdot \partial_n B + \delta_{k-2,n}d_{k-2}\left(\Gamma + \delta_{k-2,m}d_{k-2}A\right)\right] \cosh\left(\alpha_{k-2}d_{k-2}\right) + \left[\partial_n\Gamma + d_{k-2} \cdot \delta_{k-2,m} \cdot \partial_n A + \delta_{k-2,n}d_{k-2}\left(\Lambda + \delta_{k-2,m}d_{k-2}B\right)\right] \sinh\left(\alpha_{k-2}d_{k-2}\right). \tag{63}$$

$$\partial_{jmn}\gamma_{k-1} = \left[\partial_n\Lambda + d_{k-2} \cdot \delta_{k-2,m} \cdot \partial_n B + \delta_{k-2,n}d_{k-2}\left(\Gamma + \delta_{k-2,m}d_{k-2}A\right)\right] \sinh\left(\alpha_{k-2}d_{k-2}\right) + \left[\partial_n\Gamma + d_{k-2} \cdot \delta_{k-2,m} \cdot \partial_n A + \delta_{k-2,n}d_{k-2}\left(\Lambda + \delta_{k-2,m}d_{k-2}B\right)\right] \cosh\left(\alpha_{k-2}d_{k-2}\right). \tag{64}$$

And, of course, $\partial_n \Lambda = \partial_{mn} A$ and $\partial_n \Gamma = \partial_{mn} B$. These quantities are:

$$\begin{aligned}\partial_{nm} A =& D_{k-2} n_{k-2}^2 \left(\delta_{k-2,m} \partial_{jn} \beta_k + \delta_{k-2,j} \partial_{mn} \beta_k + \delta_{k-2,n} \partial_{jm} + \alpha_{k-2} \partial_{jmn} \beta_k\right) + \\ & D_{k-1} n_{k-1}^2 \delta_{k-2,m} d_{k-2} \left(\delta_{k-1,m} \partial_n \gamma + \delta_{k-1,n} \partial_m \gamma + \alpha_{k-1} \partial_{mn} \gamma_k\right).\end{aligned} \tag{65}$$

$$\begin{aligned}\partial_{mn} B =& D_{k-1} n_{k-1}^2 \left(\delta_{k-1,m} \partial_{jn} \gamma_k + \delta_{k-1,j} \partial_{mn} \gamma_k + \delta_{k-1,n} \partial_{jm} \gamma_k + \alpha_{k-1} \partial_{jmn} \gamma_k\right) + \\ & D_{k-2} n_{k-2}^2 \delta_{k-2,j} d_{k-2} \left(\delta_{k-2,m} \partial_n \beta_k + \delta_{k-2,n} \partial_m \beta_k + \alpha_{k-2} \partial_{mn} \beta_k\right).\end{aligned} \tag{66}$$

The starting values $\partial_{jmn} \beta_N$ and $\partial_{jmn} \gamma_N$ are:

$$\begin{aligned}\partial_{jmn} \beta_N =& \left[\delta_{N-1,m} \delta_{N-1,j} \delta_{N-1,n} \left(3 D_{N-1} n_{N-1}^2 + D_N n_N^2 \alpha_N d_{N-1}\right)\right] d_{N-1}^2 \cosh\left(\alpha_{N-1} d_{N-1}\right) + \\ & \Big\{ D_N n_N^2 \left[\left(\delta_{N-1,j} \delta_{N,m} + \delta_{N-1,m} \delta_{N,j}\right) \delta_{N-1,n} + \delta_{N,n} \delta_{N-1,m} \delta_{N-1,j}\right] + \\ & D_{N-1} n_{N-1}^2 d_{N-1} \alpha_{N-1} \delta_{N-1,j} \Big\} d_{N-1}^2 \sinh\left(\alpha_{N-1} d_{N-1}\right).\end{aligned} \tag{67}$$

$$\begin{aligned}\partial_{jmn} \gamma_N =& \left[\delta_{N-1,m} \delta_{N-1,j} \delta_{N-1,n} \left(3 D_{N-1} n_{N-1}^2 + D_N n_N^2 \alpha_N d_{N-1}\right)\right] d_{N-1}^2 \sinh\left(\alpha_{N-1} d_{N-1}\right) + \\ & \Big\{ D_N n_N^2 \left[\left(\delta_{N-1,j} \delta_{N,m} + \delta_{N-1,m} \delta_{N,j}\right) \delta_{N-1,n} + \delta_{N,n} \delta_{N-1,m} \delta_{N-1,j}\right] + \\ & D_{N-1} n_{N-1}^2 d_{N-1} \alpha_{N-1} \delta_{N-1,j} \Big\} d_{N-1}^2 \cosh\left(\alpha_{N-1} d_{N-1}\right).\end{aligned} \tag{68}$$

As a final remark, it must be noted that:

$$\frac{\partial^3 \alpha_k}{\partial \mu_{a,n} \partial \mu_{a,m} \partial \mu_{a,j}} = \frac{3}{8 D_k^3 \alpha_k^5} \delta_{k,n} \delta_{k,m} \delta_{k,j}. \tag{69}$$
